# National Toxic Substances Incidents Program — Nine States, 2010–2014

**DOI:** 10.15585/mmwr.ss6902a1

**Published:** 2020-03-20

**Authors:** Natalia Melnikova, Jennifer Wu, Patricia Ruiz, Maureen F. Orr

**Affiliations:** 1 Division of Toxicology and Human Health Sciences, Agency for Toxic Substances and Disease Registry, CDC

## Abstract

**Problem/Condition:**

Every year in the United States, thousands of toxic substance incidents harm workers, first responders, and the public with the potential for catastrophic consequences. Surveillance data enable public health and safety professionals to understand the patterns and causes of these incidents, which can improve prevention efforts and preparation for future incidents.

**Period Covered:**

2010–2014.

**Description of System:**

In 2010, the Agency for Toxic Substances and Disease Registry (ATSDR) initiated the National Toxic Substance Incidents Program (NTSIP), and it was retired in 2014. Nine state health departments participated in NTSIP surveillance: California, Louisiana, North Carolina, New York, Missouri, Oregon, Tennessee, Utah, and Wisconsin. The states conducted surveillance on acute toxic substance incidents, defined as an uncontrolled or illegal acute (lasting <72 hours) release of any toxic substance including chemical, biologic, radiologic, and medical materials. Surveillance focused on associated morbidity and mortality and public health actions. This report presents an overview of NTSIP and summarizes incidents and injuries from the nine participating states during 2010–2014.

**Results:**

During 2010–2014, participating state health departments reported 22,342 incidents, of which 13,529 (60.6%) met the case definition for acute toxic substance incidents, and included 6,635 injuries among 5,134 injured persons, of whom 190 died. A trend analysis of the three states participating the entire time showed a decrease in the number of incidents with injuries. NTSIP incidents were 1.8 times more likely and injured persons were 10 times more likely to be associated with fixed facilities than transportation. Natural gas, carbon monoxide, ammonia, and chemicals used in illegal methamphetamine production were the most frequent substances in fixed-facility incidents. Sodium and potassium hydroxide, hydrochloric acid, natural gas, and sulfuric acid were the most frequent substances in transportation-related incidents. Carbon monoxide was the most frequent substance in incidents with a large number of injured persons, and chemicals used in illegal methamphetamine production were the most frequent substance in incidents involving decontamination. Incidents most frequently occurred during normal business days (Monday through Friday) and hours (6:00 a.m.–5:59 p.m.) and warmer months (March–August). The transportation and warehousing industry sector had the largest number of incidents (4,476); however, most injured persons were injured in their private residences (1,141) or in the industry sectors of manufacturing (668), educational services (606), and real estate rental and leasing (425). The most frequently injured persons were members of the public (43.6%), including students. Injured first responders, particularly police, frequently were not wearing any chemically protective equipment. Respiratory system problems (23.9%) were the most frequently reported symptoms among injured persons and, in a related finding, volatilization was the most frequent type of release in incidents with injured persons.

**Interpretation:**

Industrial and transportation incidents occur frequently and have the potential for catastrophic outcomes. However, exposures to toxic substances occur frequently in other settings. Carbon monoxide, natural gas, and chemicals used in illegal methamphetamine production are commonly found in places where persons live, work, attend school, and recreate and are large contributors to incidents affecting the public. Having active NTSIP state surveillance programs did appear to improve the incidents with morbidity and/or mortality, but these programs have ended.

**Public Health Action:**

Archived NTSIP public use data are available to download from the website for analysis. There are also many publications and reports on the website to help understand chemical risks. In addition, jurisdictions might choose to collect surveillance data themselves in a similar manner to what NTSIP states did. Chemical incident surveillance data can be used by public health and safety practitioners, worker representatives, emergency planners, preparedness coordinators, industries, and emergency responders to prepare for and prevent chemical incidents and injuries. As noted by the U.S. Chemical Safety Board, more action needs to be taken to prevent large industrial incidents. Although preventing such incidents might not be in the realm of public health, describing the public health implications and preparing for them is. Another important finding of NTSIP is that industrial incidents are only part of the problem. For example, a large number of persons were injured in a private residence or vehicle (22.2%) and an educational facility (11.8%). Public health professionals must resourcefully target prevention and preparedness to protect vulnerable populations in locations where they might spend time (e.g., schools, daycares, nursing homes, recreational areas, jails, prisons, and hospitals). Reducing the threat of chemical incidents and injuries in the United States will require a concerted effort with a variety of stakeholders including industry and labor, responder groups, policymakers, academia, and citizen advocacy groups.

## Introduction

The U.S. Chemical Safety Board (CSB) is an independent, nonregulatory U.S. federal agency that conducts root cause investigations of chemical accidents at fixed industrial facilities. CSB was formed as part of the Clean Air Act Amendments of 1990 in response to the 1984 Bhopal, India disaster. The disaster resulted from a large release of methyl isocyanate from the Union Carbide Plant, which killed and permanently disabled thousands (*1*). CSB continues to investigate industrial incidents in the United States that had the potential to have similar morbidity and mortality as Bhopal. In particular, since 2015, CSB has investigated three incidents at refineries that use extremely toxic and deadly hydrofluoric acid (HF) (*2*). The most recent occurred at an oil refinery in Philadelphia, Pennsylvania, on June 21, 2019. A corroded pipe released a flammable process fluid containing HF, which formed a ground-hugging vapor cloud. The cloud ignited, causing a large fire and three large explosions that caused three separate projectiles (38,000 pounds, 23,000 pounds, and 15,500 pounds). Fortunately, the control room operator managed to pull back the bulk of the HF before the explosions. The fire was not extinguished until the following day. Although the main tank holding HF was not breached, HF was a component of the process fluid released. CSB noted no serious injuries or fatalities and will be reviewing the use of HF in refineries more closely (*3*). Although the CSB incident investigations are informative, CSB can only investigate a small portion of all incidents. CSB does not investigate nonindustrial incidents and, because it is nonregulatory, its recommendations are not enforceable.

ATSDR established the Hazardous Substance Emergency Events Surveillance (HSEES) system in 1991 and the program concluded in 2009. The HSEES system collected and analyzed information about acute releases of hazardous substances and threatened releases that resulted in a public health action, such as an evacuation in industrial and nonindustrial locations. This information was used to reduce the morbidity (injury) and mortality (death) that result from hazardous substances events experienced by first responders, employees, and the general public (*4*). In 2010, ATSDR established the National Toxic Substance Incidents Program (NTSIP), which is modeled in part after the HSEES system. NTSIP was a surveillance program that collects and combines information regarding acute toxic substances incidents from many sources to protect populations from harm (*5*). The NTSIP state incident database concluded in 2014 when funding ended; however, several states (North Carolina, Utah and Tennessee) continued to collect data on their own for a period afterwards.

This report summarizes incidents that occurred in the nine states participating in NTSIP during 2010–2014 and describes the surveillance system that was retired in 2014. Public health and safety professionals who prepare for or respond to chemical incidents can use the findings in this report to understand the patterns and causes of these incidents, thereby improving prevention efforts and preparation for future incidents and injuries.

## Methods

NTSIP was developed to replace HSEES after numerous stakeholder meetings and a HSEES program peer review. The plan was to make HSEES more nationally representative by including national incident estimates, collecting data that are more detailed on select large incidents using the Assessment of Chemical Exposures (ACE) program, and following the precautionary principle in outreach in surveillance states. The ACE program developed a modifiable toolkit to collect data after large chemical incidents. Upon request, the ACE program assembles and deploys a team to the field to implement the toolkit. The national incident estimates and ACE findings are not presented in this summary. During 2010–2012, ATSDR supported states through a competitive program announcement. During 2013–2014, as program funding decreased, ATSDR collaborated with CDC’s Public Health Associate Program to provide state assignees to support state surveillance. Nine states participated in NTSIP surveillance: California, Louisiana, North Carolina, New York, Missouri, Oregon, Tennessee, Utah, and Wisconsin (Table 1). North Carolina, Tennessee and Wisconsin reported incidents during the entire surveillance period. Prevention outreach activities were data driven and modeled after the precautionary principle, including green chemistry, inherently safer technologies, and vulnerability mapping (*5*).

**TABLE 1:** States participating in National Toxic Substance Incidents Program, by year — 2010–2014

2010	2011	2012	2013	2014
LA	LA	LA	LA	—
NC	NC	NC	NC	NC
NY	NY	NY	NY	—
OR	OR	OR	—	—
TN	TN	TN	TN	TN
UT	UT	UT	UT	UT*
WI	WI	WI	WI	WI
—	—	—	CA	—
—	—	—	MO	MO

* Includes incidents from January 1, 2014 through August 19, 2014.

### Data Sources and Collection

State health departments participating in NTSIP actively collected data from multiple sources including national (U.S. Coast Guard National Response Center and the U.S. Department of Transportation), state (departments of natural resources, departments of agriculture, divisions of emergency management, police, and bureaus of investigation), and local (poison control centers, health departments, emergency planning committees, media, and regional epidemiologists). Each state developed synergistic data-sharing agreements with the relevant organizations in their state and shared annual incident data with its stakeholders. Participating states reported to NTSIP through a web-based data portal. The goal was to enter data in a timely manner (information on at least 80% of incidents within 48 hours) so that it could be acted on if needed. Because of this requirement, entered incidents often had skeletal information and were later disqualified as additional information was collected (*5*).

### Case Definition and Variables

In state surveillance, a NTSIP case was defined as an uncontrolled or illegal acute release of any toxic substance lasting <72 hours. Toxic substances include chemical, biologic, radiologic, and medical materials. Although this is the same case definition as HSEES, the inclusion and exclusion criteria differ; therefore, the data are not similarly comparable between the two systems. Although HSEES excluded incidents where petroleum was the only substance released, NTSIP included petroleum incidents (i.e., crude oil, kerosene, gasoline, or other petroleum fuels) when a public health action (e.g., an evacuation or a health advisory) or injury occurred. NTSIP also streamlined HSEES reportable incidents to those with the most public health impact. NTSIP excluded all threatened releases, air pollution emissions (smoke stack or a flare) unless they had a public health action or injury, home incidents unless they had a public health action, and incidents involving five high frequency substances if they were under a minimum reporting quantity (paints: 100 gallons; PCBs with concentration of >50 ppm: 10 gallons; propylene or ethylene glycol: 50 gallons; and freons: 100 gallons) The minimum reporting quantity was determined by the program to be a cut point below which there was little chance for illness or injury (*5*). NTSIP captured information on adverse health effects, emergency response activities, decontamination efforts, identification of susceptible populations, and victim demographics.

The database used a geographic information system to locate nearby (within 0.25, 0.5, and 1 mile) vulnerable areas such as residences, schools, hospitals, nursing homes, licensed daycare facilities, or recreational areas (e.g., parks). Incidents were classified as transportation if they involved hazardous materials being transported by ground transportation (e.g., trucks, vans, and automobiles), railroad, aircraft, boats, ships, and pipelines outside the boundaries of a fixed facility or, in certain circumstances, on fixed-facility property. Specifically, if the event occurred on a vehicle that brought a substance to the facility before it was totally off loaded, it was coded as a transportation event. If the incident occurred while the vehicle was being loaded, it was classified as fixed-facility. All other nontransportation incidents were classified fixed-facility. Injured persons were categorized as employees, public, students, and various first-responder types (e.g., firefighter, police, and emergency medical services). For a person to be categorized as an employee, they had to be on their job. To be categorized as a student, they had to be at their school or on school transit. States selected a relevant industry code for the location of the acute toxic substance incident based on the North American Industry Classification System (NAICS), a federal standard that statistical agencies use in classifying business establishments. Personal Protective Equipment (PPE) was categorized in levels, with the highest level of protection worn being recorded. Level A is the most protective; however, it is hot and the most restrictive to movement. It includes a positive pressure full face-piece self-contained breathing apparatus (SCBA) or a positive pressure supplied air respirator with escape SCBA and a totally encapsulated chemical- and vapor-protective suit, inner and outer chemical-resistant gloves, and chemical-resistant boots. Level B has the highest level of respiratory protection but less skin protection. Level C includes a full-face respirator, chemical-resistant gloves and boots, and a hard hat. Level D is a basic work uniform. Firefighter turn-out gear is the basic work uniform that is equivalent to level D and can be worn with or without respiratory protection. Other choices of PPE included hard hats, steel toe boots, and gloves.

Chemical names were standardized and categorized. The form in which the chemical was released was recorded as volatilization (when a liquid or solid becomes a vapor after exposure to air), spills (either in liquid or solid form), fire, or explosion. Months were grouped into seasons for analysis (June, July, August = summer; September, October, November = fall; December, January, February = winter; and March, April, May = spring). Time of the day was grouped into two time blocks: nighttime (6:00 p.m. until 5:59 a.m.) and normal workday (6:00 a.m. until 5:59 p.m.).

### Analysis

Descriptive analysis was completed using SAS v 9.4. Simple frequencies and cross tabulations were completed for the incident and injured persons descriptors and for fixed facility and transportation incidents, when appropriate. In Excel, a test for trend in the number of incidents with injuries over the 5 years in the three states participating the entire time (North Carolina, Tennessee, and Wisconsin) was plotted. A fitted linear regression model with an R^2^ (a numerical value obtained by squaring the Pearson's correlation coefficient) was produced to determine the percentage of variation (from 0 to 1) explained by the relation between the dependent variable and the independent variable.

## Results

During 2010–2014, participating state health departments entered 22,342 incidents into the NTSIP system, of which 13,529 (60.6%) met the case definition. The most common reason for exclusion of entries included petroleum incidents that did not include a public health action (22.1%). For fixed-facility incidents, the most frequent primary notification sources were emergency government services (28.7%), state environmental divisions (22.2%), and the media (22.1%). The most frequent primary notification sources for transportation incidents were departments of transportation (57.9%) and emergency government services (19.9%). The three states that participated in the program the entire 5 years showed a downward linear trend (R^2^ = 0.54) in the number of incidents involving injured persons (Figure).

**FIGURE:**
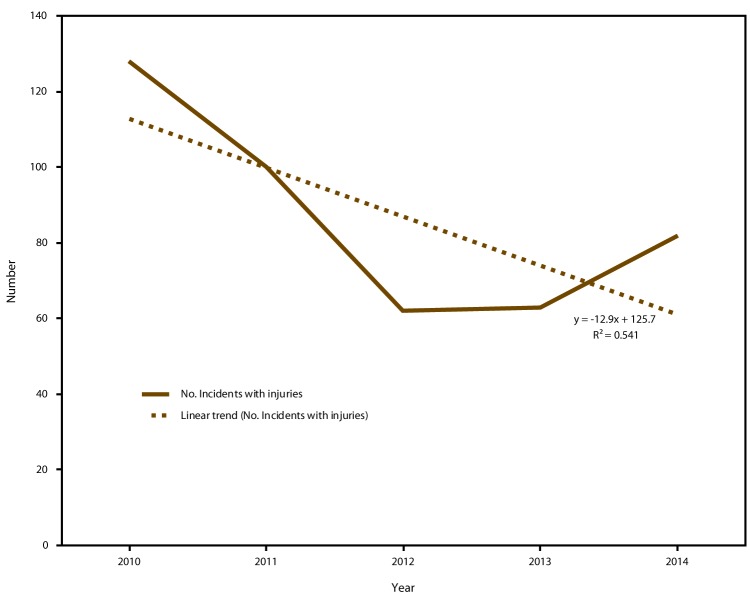
Number of incidents* with injured persons reported by three states participating the entire surveillance period^†^ — National Toxic Substance Incidents Program, 2010–2014^§^ * Total number of incidents during each year of the surveillance period: 2010 = 976; 2011 = 814; 2012 = 953; 2013 = 778; and 2014 = 864. ^†^ North Carolina, Tennessee, and Wisconsin. ^§^ R^2^ = the coefficient of determination, a statistical measure of how close to the data are to the fitted regression line.

### Incident Characterization

Almost twice as many incidents occurred in fixed facilities (8,711) than during transportation (4,818). In addition, nonfatally injured persons were approximately 10 times more frequently associated with a fixed-facility incident (4,523) than a transportation incident (421). Among reporting states, twice as many fatalities occurred in fixed-facility incidents (127) than transportation-related incidents (63). Despite only participating during 2010–2013, New York reported the most incidents with injured persons (748) and the largest number of nonfatal (2,269) and fatal (90) injured persons (Table 2).

**TABLE 2:** Incidents* with injured persons and fatalities, by type of incident and reporting state — National Toxic Substance Incidents Program, 2010–2014

State	Fixed-facility				Transportation				Total			
	No. incidents	No. incidents with injured persons	No. injured persons (nonfatal)	No. injured persons (fatal)	No. incidents	No. incidents with injured persons	No. injured persons (nonfatal)	No injured persons (fatal)	No. incidents	No. incidents with injured persons	No. injured persons (nonfatal)	No. injured persons (fatal)
California	293	34	153	2	72	8	14	**1**	**365**	**42**	**167**	**3**
Louisiana	1,881	144	262	11	833	56	75	**4**	**2,714**	**200**	**337**	**15**
Missouri	241	25	67	0	284	20	42	**4**	**525**	**45**	**109**	**4**
New York	3,378	678	2,142	66	834	70	127	**24**	**4,212**	**748**	**2,269**	**90**
North Carolina	713	122	384	12	701	50	66	**14**	**1,414**	**172**	**450**	**26**
Oregon	260	46	174	1	283	10	26	**1**	**543**	**56**	**200**	**2**
Tennessee	887	151	482	25	937	32	41	**10**	**1,824**	**183**	**523**	**35**
Utah	550	341	512	6	235	10	19	**1**	**785**	**351**	**531**	**7**
Wisconsin	508	70	347	4	639	10	11	**4**	**1,147**	**80**	**358**	**8**
**Total**	**8,711**	**1,611**	**4,523**	**127**	**4,818**	**266**	**421**	**63**	**13,529**	**1,877**	**4,944**	**190**

* An uncontrolled or illegal acute release of any toxic substance lasting <72 hours. Toxic substances include chemical, biologic, radiological, and medical materials.

More than one fourth of fixed-facility incidents (2,267 [26.0%]) resulted in an ordered evacuation compared with 303 (6.3%) evacuations for transportation incidents. For both fixed-facility and transportation incidents, more incidents occurred during the summer (4,087; mean: 44.9 per day) than spring (3,466; mean: 38.1 per day), fall (3,090; mean: 34.0 per day) and winter (2,886; mean: 32.1 per day). Most injured persons for both fixed-facility and transportation incidents were reported during the summer (1,633), followed by fall (1,232), spring (1,146), and winter (1,123). Most chemical incidents and related injuries occurred during weekdays with the greatest number on Tuesday for transportation and Wednesday for fixed-facility incidents. More incidents (8,767 [64.8%]) occurred during the normal workday than nighttime. No time period was recorded for 203 (1%) incidents.

Most transportation-related incidents occurred in three phases: 1,757 (36.5%) during unloading of a stationary vehicle or vessel, 1,212 (25.2%) while shipment was in route but not discovered until the vehicle or vessel stopped, and 1,045 (21.7%) during moving shipment. Although the largest number of incidents occurred while unloading a stationary vehicle or vessel, the largest number of transportation incidents with injuries occurred during a moving shipment (160 [60.2%]). Many of these moving vehicle injuries were a result of trauma from vehicle accidents or rollovers and not from chemical exposure. Most incidents occurred during ground transportation (4,092 [85.2%]). Railway transportation accounted for 378 (7.9%) incidents. Other incidents were related to chemical transportation by pipeline (167 [3.5%]), air (102 [2.1%]), and water (59 [1.2%]). Five incidents (0.1%) involved two different transportations modes (four rail and ground and one water and pipeline); mode of transportation was not recorded for 15 incidents.

Equipment failure was the most frequent primary contributing factor in fixed-facility incidents (4,224 [48.5%]). Specific factors cited most often for fixed-facility incidents were illicit drug production (807); improper filling, loading, or packing (509); fire (405); overspray/misapplication (298); and ruptured pipeline (233). Human error was the most frequent primary contributing factor in transportation incidents (67.5%). Specific factors cited most often for transportation incidents were improper filling, loading, or packing (1,960); forklift puncture (349); loose closure component or device (304); and vehicle or vessel derailment/rollover/capsizing (239).

Human error resulted in 2,141 (41.7%) injured persons: 1,818 (39.1%) of 4,650 persons injured in fixed-facility incidents and 323 (66.7%) of 484 injured in transportation-related incidents. Equipment failure was involved with 1,871 (36.4%) injured persons: 1,798 (38.6%) for fixed-facility incidents and 73 (15.1%) for transportation-related incidents.

The majority of incidents (9,694 [71.7%]) did not have a secondary contributing factor. Among incidents for which a secondary contributing factor was reported, equipment failure (1,574 [11.6%]) was most frequently listed. The most frequently reported areas with vulnerable populations within 0.25 mile were residences (10,996) and daycare centers (1,701).

### Chemicals

Of the 8,711 incidents reported in fixed facilities, 7,990 (91.7%) involved a single chemical. Natural gas (14.1%), carbon monoxide (8.4%), illegal methamphetamine production chemicals (6.9%), and ammonia (6.7%) were the chemicals primarily involved in fixed-facility incidents (Table 3).

**TABLE 3:** Most common chemicals released in fixed-facility incidents — National Toxic Substance Incidents Program, 2010-2014

Chemical	No. (%)*
**Natural Gas**	1,127 (14.1)
**Carbon Monoxide**	672 (8.4)
**Illegal Methamphetamine Production Chemicals**	554 (6.9)
**Ammonia**	536 (6.7)
**Mercury**	257 (3.2)
**Sulfuric Acid**	236 (3.0)
**Alkaline Hydroxide (sodium and potassium)**	227 (2.8)
**Propane**	224 (2.8)
**Chlorine**	203 (2.5)
**Hydrochloric Acid**	167 (2.1)

* Percentages calculated using total number of single chemical fixed-facility incidents (N = 7,990) as the denominator.

Of the 4,818 transportation-related incidents, 4,689 (97.3%) involved a single chemical. Alkaline hydroxides (sodium hydroxide or potassium hydroxide) accounted for the highest percentage of transportation-related incidents (10.2%), followed by sulfuric acid (5.5%) hydrochloric acid (4.8%), and natural gas (3.4%) (Table 4). Most injuries involved a single chemical incident (4,761 [92.7%]), and most fatalities were from a single chemical incident (175 [92.1%]).

**TABLE 4:** Most common chemicals released in transportation incidents — National Toxic Substance Incidents Program, 2010-2014

Chemical	No. (%)*
**Alkaline Hydroxide (sodium and potassium)**	479 (10.2)
**Sulfuric Acid**	258 (5.5)
**Hydrochloric Acid**	224 (4.8)
**Natural Gas**	160 (3.4)
**Hydrogen Peroxide**	124 (2.6)
**Acetone**	117 (2.5)
**Flammable Liquid NOS**	88 (1.9)
**Resin NOS**	77 (1.6)
**Isopropanol NOS**	75 (1.6)
**Ink**	59 (1.3)

**Abbreviation**: NOS = not otherwise specified.

* Percentages calculated using total number of single chemical transportation incidents (N = 4,689) as the denominator.

Volatilization was involved in almost half (47.5%) of fixed-facility incidents, followed by spills (37.7%) and multiple release types (11.9%). Fires and explosions accounted for less than 1% each of fixed-facility incidents. Spills were involved in many more (85.2%) transportation incidents followed by volatilization (8.7%) and multiple release types (5.3%). Transportation incidents included 14 (0.2%) fires and seven (0.1%) explosions. Volatilization was involved in 2,766 (53.9%) injuries and 86 (45.3%) deaths.

### Substances Causing the Most Injuries

Almost half (46.8%) of the 5,134 reported injured persons attributed injuries to seven hazardous substances: carbon monoxide (1,251), natural gas (293), illegal methamphetamine production chemicals (a category used when the individual involved substances could not be identified) (248), chlorine (193), sulfuric acid (159), propane (145), and ammonia (113). The properties of these chemicals make them particularly hazardous.

In 12 incidents, ≥30 injuries occurred. These 12 incidents were in areas where many persons gathered, including some vulnerable populations (e.g., children, elderly, or incarcerated), and where a chemical release was unexpected. Seven of the incidents involved carbon monoxide exposure. For example, at an elementary school, a water heater exhaust pipe became disconnected, venting gas into a mechanical room, kitchen, and classrooms. Approximately 280 students were at the school at the time. An evacuation was ordered, 30 persons were treated at the scene, and 44 students and teachers were taken to local hospitals and treated for carbon monoxide poisoning. Another carbon monoxide exposure incident was reported in a detention center where a malfunctioning gas-powered water heater sent carbon monoxide through the ventilation system into cellblocks, requiring an evacuation and sickening 47 inmates, who reported nausea and headache.

Of the 5,134 injured persons (fatal and nonfatal), most (49.4%) were treated at a hospital but not admitted. The public and students at school accounted for more than half (51.2%) of all injured persons. Other categories of injured persons included employees (37.6%) and first responders (9.3%). Among injured first responders, the most frequently injured type were career (paid) firefighters and police officers, and least injured were company response team members or third party clean up contractors. Firefighters, both career and volunteer, were more likely to be wearing firefighter turn-out gear, either with or without respiratory protection, and all other first responders (i.e., police officers, not specified responders, emergency medical services, and hospital personnel) were more likely to be wearing no PPE.

Of the 3,085 injured persons whose sex was reported, more than twice as many were male (2,104) than female (981), and the proportion of affected varied by category. More male than female employees were injured (912 versus 254). The number of injured males also was noticeably higher than females among first responders (297 versus 23) and less so in the public (893 versus 704). Of the 4,090 injured persons for whom age category was reported, 790 (19.3%) were aged <18 years.

Each person could have multiple reported adverse health effects. More than two thirds (3,465 [67.5%]) had only one reported adverse health effect, 989 (19.3%) had two, 312 (6.1%) had three, and 59 (1.1%) had more than three. A total of 304 (5.9%) had no reported health effects.

Respiratory system problems (25.8%), dizziness or other central nervous system problems (18.5%), burns (10.2%), and headache (9.4) were the most commonly reported adverse health effects. Respiratory system problems (26.7%) were most frequent in fixed-facility incidents and trauma (39.8%) in transportation incidents. Nonchemical-related traumas (e.g., from a vehicle crash or explosion debris) (328) were more common than chemical-related traumas (84) (Table 5).

**TABLE 5:** Number and characteristics of adverse health effects, by type of incident* — National Toxic Substance Incidents Program, 2010–2014

Characteristic	Incident type		
	Fixed facility	Transportation	Total
	No. (%)	No. (%)	No. (%)
Respiratory	1,629 (26.7)	85 ( 16.0)	**1,714 (25.8)**
Dizziness or other CNS^†^	1,180 (19.3)	47 (8.9)	**1,227 (18.5)**
Burns	598 (9.8)	76 (14.3)	**674 (10.2)**
Chemical related	192 (32.1)	38 (50.0)	**230 (34.1)**
Thermal-related^§^	293 (49.0)	32 (42.1)	**325 (48.2)**
Both types	73 (12.2)	6 (7.9)	**79 (11.7)**
Unknown type	40 (6.7)	0 (0.0)	**40 (5.9)**
Headache	604 (9.9)	17 (3.2)	**621 (9.4)**
Gastrointestinal	560 (9.2)	9 (1.7)	**569 (8.6)**
Eye irritation	469 (7.7)	27 (5.1)	**496 (7.5)**
Trauma	264 (4.3)	211 (39.8)	**475 (7.2)**
Chemical-related	74 (28.0)	10 (4.7)	**84 (17.7)**
Nonchemical-related^§^	150 (56.8)	178 (84.4)	**328 (69.1)**
Both types	13 (4.9)	16 (7.6)	**29 (6.1)**
Unknown type	27 (10.2)	7 (3.3)	**3 (7.2)**
Skin irritation	191 (3.1)	15 (2.8)	**206 (3.1)**
Short of breath	163 (2.7)	6 (1.1)	**169 (2.5)**
Heart problems	72 (1.2)	7 (1.3)	**79 (1.2)**
Heat stress	62 (1.0)	1 (0.2)	**63 (0.9)**
Other	313 (5.1)	29 (5.5)	**342 (5.2)**
**Total** ^¶^	**6,105 (100.0)**	**530 (100.0)**	**6,635 ( 100.0)**

**Abbreviations**: CNS = central nervous system.

* An uncontrolled or illegal acute release of any toxic substance lasting <72 hours. Toxic substances include chemical, biologic, radiological, and medical materials.

^†^ Other CNS problems includes fainting, passing out, lightheadedness, ataxia, numbness, tingling, and twitching.

^§^ Nonchemical related traumas and thermal burns might be related to a fire, explosion, or a traffic accident rather than from exposure to a chemical.

^¶^ Adverse health effects for 304 injured persons were missing.

### Response Actions

Of the 4,610 injured persons (fatal and nonfatal) with known decontamination status, most were not decontaminated (3,748 [81.3%]). Among those who were decontaminated, 432 (9.4%) were decontaminated at the scene of the incident and 345 (7.5%) at a medical facility. For 85 (1.8%) injured persons, decontamination occurred both at the scene of the incident and at a medical facility.

The majority (10,180 [75.2%]) of incidents did not require a public health action, despite the fact that some incidents (petroleum, home, and pollution incidents) required a public health action to meet the case definition. Fixed-facility incidents required more public health actions than did transportation-related incidents (2,664 [30.6%] versus 227 [4.7%]). The most frequent public health action for fixed facilities and transportation was environmental sampling (2,221 and 173 incidents, respectively). Other health actions included health investigations (six), health advisory (five), use of an alternative water source (one), well survey (one), and shut down of water intakes (one).

A single type of responder responded to 7,639 (56.5) incidents and multiple types of responders responded to 5,062 (37.4%) incidents. For 566 (4.2%) incidents, no responders were involved. Responder information was missing for 262 (1.9) incidents. When there was only one responder type (7,639), a company’s response team was most often there for transportation incidents (49.1%) and fixed-facility incidents (24.2%). The fire department also frequented fixed-facility incidents (20.5%).

During 2010–2014, a total of 2,570 (18.9%) incidents resulted in an evacuation order, and 249 (1.8%) incidents resulted in a shelter-in-place order. In most incidents requiring an evacuation order (1,612 [62.7%]), ≤50 persons were evacuated. Although spills (liquid or solid) were much more frequent than other types of releases, evacuation orders were much more frequent when there was a vapor (36.2%), a fire (35.5%), or an explosion (36.6%) than a spill (8.4%).

### Industry

During 2010–2014, the largest number of NTSIP-eligible incidents (4,476 [33.1%]) was attributed to the transportation and warehousing sector (NAICS codes 48 and 49) (Table 6). This sector includes transportation by air, rail, water, truck, and transit as well as ground passenger transit, pipeline transport, scenic and sightseeing transport, transportation support activities, and postal service and courier transport. The second largest number of NTSIP-eligible incidents was in the manufacturing sector (NAICS codes 31, 32, and 33) (2,365 [17.5%]). Most (1,855) occurred in NAICS code 32, which includes printing and associated activities and the manufacture of wood products, paper, printing, petroleum and coal, chemical, plastic and rubber, and nonmetallic minerals. Manufacturing incidents resulted in the greatest number of injures (668 [13.0%]), followed by educational services (NAICS code 61) (606 [11.8%]) and real estate and rental leasing (NAICS code 53) (425 [8.3%]) (Table 6). Although transportation and warehousing incidents were the most frequent, they resulted in fewer injured persons than other categories. These data are consistent with findings that fixed-facility incidents were more likely to result in a greater number of injured persons.

**TABLE 6:** Type of industries, by incidents, injured persons, and fatalities — National Toxic Substance Incidents Program, 2010–2014

NAICS Code	Incidents	Injured persons	Fatalities^†^
	No. (%)	No. (%)	No. (%)
(11) Agriculture, Forestry, Fishing, and Hunting	232 (1.7)	57 ( 1.1)	3 (1.6)
(21) Mining	117 (0.9)	25 (0.5)	1 (0.5)
(22) Utilities	823 (6.1)	58 (1.1)	5 (2.6)
(23) Construction	147 (1.1)	81 (1.6)	2 (1.1)
(31–33) Manufacturing	2,365 (17.5)	668 (13.0)	10 (5.3)
(42) Wholesale Trade	397 (2.9)	117 (2.3)	6 (3.2)
(44–45) Retail Trade	295 (2.2)	192 (3.7)	7 (3.7)
(48–49) Transportation and Warehousing	4,476 (33.1)	247 (4.8)	13 (6.8)
(51–52) Information, Finance, and Insurance	43 (0.3)	94 (1.8)	1 (0.5)
(53) Real Estate and Rental and Leasing	591 (4.4)	425 (8.3)	8 (4.2)
(54) Professional, Scientific, and Technical Services	78 (0.6)	50 (1.0)	1 (0.5)
(55) Management of Companies and Enterprises	3 (0.0)	3 (0.1)	0 (0.0)
(56) Administration, Waste Management, and Remediation Services	207 (1.5)	54 (1.1)	1 (0.5)
(61) Educational Services	328 (2.4)	606 (11.8)	1 (0.5)
(62) Health Care and Social Assistance	193 (1.4)	211 (4.1)	5 (2.6)
(71) Arts, Entertainment, and Recreation	93 (0.7)	188 (3.7)	0 (0.0)
(72) Accommodation and Food Services	204 (1.5)	285 (5.6)	4 (2.1)
(81) Other Services	177 (1.3)	134 (2.6)	4 (2.1)
(92) Public Administration	145 (1.1)	161 (3.1)	7 (3.7)
(A8) Not an Industry*	569 (4.2)	144 (2.8)	17 (8.9)
(A9) Unknown*	351 (2.6)	193 (3.8)	2 (1.1)
(VR) Private vehicle or residence*	1,695 (12.5)	1,141 (22.2)	92 (48.4)
**Total**	**13,529 (100.0)**	**5,134 (100.0)**	**190 (100.0)**

**Abbreviation:** NAICS = North American Industry Classification System.

* Code defined by National Toxic Substance Incidents program.

^†^ Fatalities are a subset of number of injured persons.

Several states have used their NTSIP program to better prepare for chemical emergences. For example:

Oregon NTSIP staff worked with preparedness officials in Columbia County, which has the most ammonia releases in Oregon, to initiate a public education campaign about anhydrous ammonia and sheltering-in-place and developed a preparedness fair and several basic shelters in place (*4*).
New York NTSIP contributed to swimming pool chemical incident awareness by developing seven fact sheets on pool chemical safety (https://www.health.ny.gov/environmental/chemicals/pool_chems) that were widely distributed to private and public pool and spa operators. New York NTSIP also prepared tips about swimming pool chemical safety for CDC’s Recreational Water Illness and Injury Prevention Week, May 19–25, 2014. CDC used the messages in “Have You Heard?” bulletins, which displayed activities by state and local partners to promote pool safety and prevent injuries (*4*).
Louisiana NTSIP monitored incidents and informed responders during hazmat situations. For example, a valve released sulfuric acid at a major petrochemical plant in St. Charles Parish, Louisiana, and two employees became nauseated. Employees were evacuated and air monitoring was conducted until the site was secure. To protect vulnerable populations, Louisiana NTSIP staff created emergency response maps that showed the spill location in relation to hospitals, daycare centers, and other facilities of interest and fact sheets on sulfuric acid (*4*).
Wisconsin NTSIP staff developed mapping software to identify vulnerable populations and better prepare them for hazmat incidents. The software included transportation corridors; chemical spills; chemical storage data; population demographics; and locations of schools, universities, nursing homes, and hospitals. To test the software, one county health department identified a recreational complex that serves 800,000–1,000,000 persons annually that was located less than 300 feet from an Amtrak rail line and less than 1.5 miles from two chemical processing plants and a major regional electric power generating station. NTSIP staff and partners from other state agencies conducted a tabletop exercise at that complex simulating a freight train derailment and release of acrylonitrile. The exercise identified areas for improvement that included appointing a liaison from the responder community to work with the complex on a regular basis to build rapport. Improvements were undertaken to make the complex less vulnerable if a chemical event occurs nearby (*4*).
ATSDR staff collaborated with state staff to produce special analysis of data for publication or presentations. Topics included carbon monoxide incidents, school incidents, first responder injuries, chemical suicides, methamphetamine laboratory incidents, secondary contamination, swimming pool incidents, safety of chlorine and its alternatives, chemical bombs, and chemical incidents during hurricanes (https://www.atsdr.cdc.gov/ntsip/publications.html).

## Public Health Implications

Reform and modernization of chemical safety regulation often comes in a reactive manner. Following the Bhopal disaster, CSB was formed (*1*) and ATSDR began its HSEES chemical incident surveillance (*4*). In addition, numerous safety regulations were enacted and an industry-led process safety movement was started. After a long period with no large incidents, less attention was focused on safety habits and regulations. A catastrophic incident on April 17, 2013, in West Texas created renewed interest in safety and prevention strategies as evidenced by the Executive Order (Improving Chemical Facility Safety and Security) requiring federal agencies to modernize and improve chemical process safety. Nonetheless, this renewed attention to chemical process safety and reform was not sufficient to prevent the June 21, 2019 HF incident in Philadelphia (*3*) that, according to the company’s Risk Management Plan, could have affected over a million persons (*6*). Because of continuing incidents like this, CSB believes that more action must be taken by industry to prevent a catastrophic incident like Bhopal from occurring in the United States. Preventive measures include more attention to process safety management and use of safer alternatives for extremely hazardous substances like hydrogen fluoride (*3*).

Environmental health legislation, one preventive measure, can be spurred by a noteworthy incident in a community. For example, Pennsylvania lawmakers recently proposed banning HF in refinery operations after the toxic chemical was released in a refinery explosion in 2019 (*7*). Two other refineries in the region use HF, but the proposed ban would not apply to them. Also, a similar effort in previous years to ban HF in refineries in Southern California after an incident there was unsuccessful (*7*). Public health agencies can help prevent morbidity and mortality by proactively collecting surveillance data to support and prioritize safety and preventive measures in their jurisdictions. NTSIP public use data are available for download on the NTSIP webpage along with reports and journal articles. The substances most frequently released or that cause most injuries in fixed facilities are hazardous chemicals that the general public can be exposed to where they live, work, attend school, and recreate (e.g., natural gas, carbon monoxide, chemicals used in illegal methamphetamine production, and ammonia). Public health professionals must resourcefully target prevention and preparedness to protect vulnerable populations in locations where they might spend time (e.g., schools, daycare centers, nursing homes, recreational areas, jails, prisons, and hospitals). Reducing the threat of chemical incidents and injuries in the United States will require a concerted effort with a variety of stakeholders including industry and labor, responder groups, policymakers, academia, and citizen advocacy groups.

First responders would benefit from additional attention to chemical safety. They are often injured in chemical incidents and are not always wearing PPE. One study found that many first responders were injured in chemical incidents while responding to illegal methamphetamine laboratories (*8*). First responders might be exposed to a diverse array of chemicals. Fentanyl and its analogs and the growing chemical suicide trend (*9*,*10*) are new exposures that surveillance helps characterize and prevent. NIOSH provides resources for respirator selection (*11*) and has developed the Emergency Responder Health Monitoring and Surveillance framework for health monitoring of responders in the predeployment, deployment, and postdeployment phases (*12*).

## Limitations

The findings in this report are subject to at least three limitations. First, NTSIP states used differing data sources and reporting procedures to complete the incident form; therefore, aggregated data across states and across incidents should be interpreted with caution. Second, NTSIP does not capture all incidents that occur because many incidents are only reported if they have an injury or public health action. Finally, incidents that occurred in the transportation and warehousing industries often are associated with motor vehicle crashes, and injuries from those incidents might be related to the trauma of the crash rather than the chemical release.

## Conclusions

State chemical incidents surveillance provided important information, and lessons learned from these data can guide future prevention and preparation for chemical incidents. ATSDR continues to support state and local health departments in learning about chemical incidents through the ACE program. ACE has a modifiable toolkit to preform incident investigations and can provide expert technical assistance, deploying to the field if needed. ACE training will be offered at no cost to state and local staff at the Federal Emergency Management Agency Center for Domestic Preparedness in Anniston, Alabama, beginning in 2020. Additional information on state surveillance activities, the ACE team, the national database estimates, or any of the programs’ resources and updates is available at www.atsdr.cdc.gov/ntsip.
